# Mental Health Service Users’ Perceptions of Stigma, From the General Population and From Mental Health Professionals in Mexico: A Qualitative Study

**DOI:** 10.1007/s10597-020-00706-4

**Published:** 2020-09-05

**Authors:** Emmeline Lagunes-Cordoba, Alan Davalos, Ana Fresan-Orellana, Manuela Jarrett, Jorge Gonzalez-Olvera, Graham Thornicroft, Claire Henderson

**Affiliations:** 1 Health Service and Population Research Department, Institute of Psychiatry, Psychology and Neuroscience, King’s College London, De Crespigny Park, London, SE5 8AF UK; 2 Instituto Nacional de Psiquiatría “Ramón de La Fuente Muñíz”, Mexico City, Mexico; 3 School of Health Sciences, University of London, London, UK; 4 Comisión Nacional para la Prevención de Adicciones, Mexico City, Mexico; 5 Centre for Global Mental Health and Centre for Implementation Science, Institute of Psychiatry, Psychology and Neuroscience, King’s College London, London, UK; 6 Centre for Implementation Science, Institute of Psychiatry, Psychology and Neuroscience, King’s College London, London, UK

**Keywords:** Stigma, Discrimination, Attitudes, Psychiatrists, Service users

## Abstract

Negative attitudes towards people with mental health disorders have been widely studied and identified in the general population, and even within health care professionals. Moreover, studies focused on service users have also identified mental health professionals, including psychiatrists, as a source of stigma. However, in Mexico and Latin America few studies have been conducted addressing this issue. To explore mental health service users’ perceptions of stigma by members of the general population and by psychiatrists in Mexico, service users at a psychiatric hospital in Mexico were invited to participate in either focus groups or individual interviews, which were audio recorded, transcribed and analysed using thematic analysis. A total of 47 service users participated in this study. The results suggested that participants were not only aware of the possible consequences of mental health related stigma, but they have also experienced stigmatisation for having a mental illness. Participants also considered psychiatrists can hold negative attitudes towards people with mental illness, something that can represent a barrier for them to have optimal quality of care. Therefore, participants agreed that these attitudes should be addressed to improve the care they received from these professionals. This study suggests that, like members of the general population, psychiatrists are also considered as a source of stigma by people with mental illness in Mexico. These findings not only add to previous work conducted in Mexico and other countries, they also confirm the importance of addressing negative attitudes in this group of health professionals.

## Introduction

Mental health service users often deal with both, the effects of mental disorders and the emotional and behavioural reactions of others towards them. Research has found that social distance, stereotyping, lack of empathy and patronising attitudes are some of the most common experiences people with mental disorders suffer from the general population (Schulze and Angermeyer 2003; Dinos et al. 2004; Thornicroft et al. 2009).

Family members and close friends are among those commonly considered as the main source of stigma in these studies. However, people with mental disorders also consider health care professionals as sources of stigma and discrimination (Schulze and Angermeyer 2003; Thornicroft et al. 2009; Henderson and Thornicroft 2013; Hamilton et al. 2014; Otewell 2016). Some studies have shown that health care staff often have limited mental health knowledge; hold negative attitudes towards people with mental illness; reject or make people with certain diagnoses wait longer; and dismiss and misattribute physical symptoms to patients’ mental disorders, which often leads to under-diagnosis and delay in accessing treatments (Thornicroft et al. 2007; Knaak et al. 2017).

Similarly, studies focused on mental health professionals have found evidence of stigmatising attitudes and behaviours (Jorm et al. 1999; Hugo 2001; Lauber et al. 2006; Nordt et al. 2006; Loch et al. 2013; Gras et al. 2014). Other studies have also shown these professionals have more pessimistic views about recovery than the general population (Jorm et al. 1999; Hugo 2001; Schulze 2007; Thornicroft et al. 2010). This might be explained by a phenomenon known as “physician’s bias”, caused by their lack of contact with fully recovered patients (Thornicroft et al. 2007, 2010).

Service users also report feeling stigmatised by mental health professionals, manifested by a lack of interest in their personal or their mental health history; diagnoses given with a negative prognosis and without empathy (Schulze and Angermeyer 2003; Schulze 2007; Horsfall et al. 2010); involuntary disclosure of their illness due to medication side effects; lack of information they receive about their diagnoses or treatments; being treated as children; and being excluded from important decisions (Schulze 2007; Thornicroft et al. 2007).

In Mexico, a middle-income country (Wang et al. 2007), with relatively high prevalence of mental disorders (Medina-Mora et al. 2003), a low proportion of people seek mental health care and even fewer receive care. This problem is believed to be caused, among other factors, by social stigma (De la Fuente and Heinze 2014).

Little research has been conducted in Mexico regarding these issues, and most research has focused on public stigma (Mora-Rios et al. 2013a, 2013b; Robles-Garcia et al. 2013), or on the attitudes of medical and psychology students (Fresan et al. 2012, 2013; Vargas-Huicochea et al. 2017). Still, we identified one study which focused on service users’ perspectives, that reported users considered health care personnel as the second most important source of stigma and discrimination, preceded only by family members (Mora-Rios and Bautista 2014).

The aim of this study is to explore mental health service users’ perceptions of stigma from the general public, but more importantly, from psychiatrists in Mexico, with the ultimate goal of developing a targeted anti-stigma intervention for Mexican psychiatric trainees.

## Methods

### Design and Setting

This qualitative study used focus groups and individual interviews with service users attending a psychiatric hospital in Mexico City, which provides psychiatric treatment to people with any mental disorder with mental capacity. The study was approved by the Psychiatry, Midwifery and Nursing Research Ethics Committee of King’s College London and from the Research Ethics Committee of the hospital where this research was conducted.

### Participants

All participants were outpatients; over 18 years old; treated by psychiatric trainees or psychiatric consultants and managed in either the general adult psychiatry service or in the different clinics of the hospital (i.e. eating disorder clinic, old age psychiatry). We included individuals with any diagnosis that met the inclusion criteria and were willing to participate. This participant sample was chosen based on theoretical sampling, which facilitates identifying diverse possibilities that are theoretically relevant to our research questions; for example we aimed to include patients from both genders, with different psychiatric diagnoses and treated by trainees and consultants from the different specialty clinics. This sample strategy also allowed for theoretical saturation, meaning that individual’s recruitment ended when the ability to obtained new information was reached (Guest et al. 2006).

### Recruitment and Data Collection

We invited service users through posters displayed in the hospital. Interested participants were able to leave their details with waiting room assistants who provided them with information sheets and consent forms. We also invited participants of existing therapy groups from the following clinics: eating disorders, obsessive compulsive disorders, old age psychiatry, substance abuse disorders and borderline personality disorder. There were no drop outs; however, two participants had to leave focus groups early due to other commitments.

All focus groups were conducted by one of us (ELC) and co-facilitated by another member of our research group (AD). Focus groups and individual interviews were conducted in meeting rooms at the hospital. We moderated every discussion using opened-ended questions from a topic guide, which included questions addressing personal experiences of stigma and discrimination form members of the general population, experiences of stigma from mental health providers and possible solutions to reduce stigma. A copy of the topic guide is available upon request.

### Data Analysis

We transcribed and analysed all interviews in Spanish, using thematic analysis with both, inductive and deductive approach at a semantic level, as the coding process initially involved identifying codes aimed to answer our research questions, but an inductive process was later followed to establish new categories and themes (Braun and Clarke 2006; Roberts et al. 2019). Coding was performed by grouping together units of text focused on the same matter (Graneheim and Lundman 2004). Data analysis was first conducted by one of us (ELC) and reviewed by another researcher (MJ) for credibility, later researchers discussed and compared identified codes, themes and sub themes until agreement was reached. Themes and subthemes were later reviewed, modified, rejected or replaced by consensus and by comparing them with the original data set. A total of 73 codes were grouped into categories; these were arranged into subthemes, which were then organised into three overarching themes. Each subtheme was defined and refined to identify each theme and make sure they were organised and categorised in a coherent way. Categories, subthemes and quotes used to exemplify these themes were translated to English. See Table 1 for examples of trigger questions, and the resulting categories, subthemes and themes.

**Table 1:** Categories, Subthemes and themes emerged from trigger questions

Example of trigger questions	Categories	Subthemes	Themes
How do you think people react to psychiatric patients?	Social distance	Reactions from members of the general population	Experiences of stigma from the general population
	Fear		
	Paternalism		
Which experiences have you had from people’s reactions towards yourself?	Discrimination		
	Lack of empathy		
	Labelling and judgement		
What has changed since your diagnosis?	Concealment	Reactions to having a mental disorder	
How have you responded to other reactions?	Emotional reactions		
	Self-stigma		
	Aggression		
Overall, how has been your relationship with them (psychiatrists)?	Cold/distant treatment	Perceptions of stigmatising attitudes in psychiatrists	Perceptions of stigma from psychiatrists
	Lack of clear explanations		
	Focus on pathology or medication		
Have you ever feel uncomfortable with anything your doctor has done or said?	Labelling		
Why do you think they (psychiatrists) behave in this way?	Desensitisation	Causes of stigmatising attitudes in psychiatrists	
	Burnout		
	Lack of training		
Are there any other reasons that could explain why psychiatrists behave in this way?	Change of psychiatrists		
	Unrealistic expectations		
Do you think they (psychiatrists) also need some kind of program to reduce stigma?	Raising awareness	Proposed methods to improved attitudes in psychiatrists	Interventions to improve attitudes in psychiatrists
What do you think are the best strategies to reduce stigma in psychiatrists?	Formal training		

Trustworthiness was preserved by evaluating credibility, dependability and transferability (Graneheim and Lundman 2004). Credibility was established by theoretical sampling until theoretical saturation was reached, and by seeking consensus regarding themes with a second researcher. Dependability, which refers to the degree that data change over time as a result of the researcher’s decisions during the process, was assessed by detailing the recruitment process and interviews procedures, and by reflecting on how authors’ experiences could have influenced the interviews and the development of themes. Finally, transferability, which refers to the extent findings can be applicable to other settings, was not assessed, as this study was conducted in one place so it is not possible to be confident about the transferability of the results. However, we provided a wide description of the research context, participants’ characteristics, data collection and quotations of all the themes described, so readers can consider the extent to which findings may be transferable to a different context.

## Results

We conducted nine focus groups, with a minimum of two and a maximum of 11 participants; and two individual interviews. A total of 47 people participated, 39 females and 8 males. Groups lasted 20 to 100 min. Interviews lasted 10 min to 25 min. Focus groups and interviews were recorded using an audio-recorder. There was no debrief with participants, but the information sheet included a statement saying that if they would like to know the results of the study, they could contact the principal researcher. Only one participant got in contact to learn about the results. We identified five subthemes from 21 categories, which were grouped into three overarching themes: Experiences of stigma from the general population; perception of stigma from psychiatrists; and interventions to improve attitudes in psychiatrists.

### Experiences of Stigma From the General Population

Most participants recognised and provided several examples in which they felt they were stigmatised by members of the general population. Within this theme, there were two subthemes identified, *reactions from members of the general population* and *reactions to having a mental disorder*, each one with categories described.

#### Reactions From Members of the General Population

##### Social Distance and Fear

Most participants considered members of the general population tend to reject or avoid people with mental illness, potentially due to fear of their illness, reactions that have caused them to lose meaningful relationships like close friends or even partners.

As much as they love us and care about us, they move away, many of them say that we have nothing, and the ones that are helping us get tired and start to be aggressive… then they reject you.

I feel people are like… ‘there she comes, everybody run, hide, because what if she gets a crisis, she might attack us’… I feel that has been very bad for me.

##### Paternalism and Discrimination

Participants mentioned that they are commonly treated by others as if they are incapable or unable to take care of themselves, while some others felt that these negative assumptions were associated with discriminatory behaviours.

There is no trust, even if you are fine. I told them I wanted to get independent and live by myself, so they came to speak with the psychiatrists to ask if it was logic that a woman with my disorder (borderline personality) could live alone and be independent. They don’t believe in me, is very uncomfortable.

They fired me because of that, for having a crisis…. because what a coincidence, I had a crisis and one or two days after, no! you are no longer fit for your job, then out.

##### Lack of Empathy

Many participants considered that most people do not seem to understand their mental disorders, as they tend to minimise their symptoms and even accuse participants of making up their symptoms. Some participants also mentioned some people do not believe mental disorders even exist. The use of “motivational” phrases, which participants described as phrases others use to cheer them up or to show support, was also considered as an example of lack of empathy, as participants reported feeling misunderstood and even dismissed when people use those phrases.

Most members of my family don’t believe that these things are something real or valid, it’s like, you are exaggerating, you don’t need it, you should be able to overcome it by yourself.

There is no support from people near to you and to be honest, I already hate the phrase ‘échale ganas’ (try your best/make an effort).

##### Labelling and Judgement

The use of negative labels and judgement were also among the most common responses in the general population reported by most participants. The labels “crazy” and “mentally ill” were the most commonly reported.

My ex-husband never stopped saying I was crazy, all his family said: ‘she is crazy, she is dangerous’. There was even a point when my son also started telling me: … mom you are ill, you are crazy.

#### Reactions to Having a Mental Disorder

Reactions to having a mental disorder was identified as a subtheme, but reactions reported by most participants were often related to others’ negative attitudes towards them. Most participants reported they often feel they need to conceal their mental disorder to avoid possible negative reactions, and those who were not able to conceal their illness, seemed be more likely to internalise negative assumptions about people with mental illness or to consider true what others say about them. We categorised these as c*oncealment* and s*elf-stigma.*

…it is not something you would like to yell to the world, it is something you want to keep to yourself because you never know how people would react.

They have called me crazy, damaged of my head… all that make me feel like, could I or could I not be crazy?

*Emotional reactions* and *aggression* Participants also report that they were more likely to emotionally react to others’ lack of empathy and avoidance by feeling sad and isolated, or even by being aggressive towards both, other people or themselves.

I did mention it to my siblings, and the truth is that they do point at you saying, ‘we told you that you were crazy… we saw there was something wrong with you’. That makes you feel very upset… You feel worse.

…when they start telling me that it’s only emotional blackmail, I first start crying… Then I would go to my room and start hitting things… I used to hurt myself, I felt so much aggression that I needed it to take it out. Once I broke a window… I feel I just reacted….

### Perceptions of Stigma From Psychiatrists

Although most participants said that overall, their experiences with psychiatrists were positive, many participants reported having experienced or witnessed a situation which they considered was linked to stigma. This category was divided into two subthemes: *perceptions of stigmatising attitude in psychiatrists* and *causes of stigma in psychiatrists*.

#### Perceptions of Stigmatising Attitudes in Psychiatrists

##### Cold and Distant Treatment and Lack of Clear Explanations

The most common complaint from participants was feeling that psychiatrists are often cold and distant, as if there was a barrier between them that does not allow them to communicate well. Also, some participants commented that they are still not sure which is their diagnosis or their implications. Others complained about not receiving enough information about their treatment, like the reasons for a given medication, or the expected length of their treatment.

…there is a big barrier between the psychiatrist and the patients, I mean, while I’m talking about the deepest and most heart-breaking parts of my existence, they just write… as a patient I have sometimes felt misunderstood, like there is no link.

I have been through several diagnoses, it was major depression, then some eating disorder, I don’t remember which one but it was something like bulimia, then it was a sleeping disorder, and then I landed in the Borderline personality disorder. I really got lost between all these diagnoses I received.

##### Focus on Psychopathology/Medication and Lack of Interest in the Personal History

One of the themes emerged was the little interest psychiatrists had in patients’ personal lives, focusing mostly on their psychiatric illness and in adjusting medication. Although comments related to lack of interest in their personal history were not that common, some participants stated that when psychiatrists do not read their medical records or ask repetitive questions, they feel psychiatrists are not really interested in them.

One doctor told me that they are not there for us to tell them our problems, that psychologists were there for that. That psychiatrists are only there for medication… I told him: why are you so cold? And he said: ‘we are not here to fix or to listen to your problems, we are here only to medicate you.

Many times they don’t even have your medical records, and even if they have them, it’s always: ‘what’s your diagnosis? How much do you take of this? How have you felt? Bad, OK, then I’m going to change your dose’. They don’t even know what’s wrong with you; if we only go for them to give us medication then they are not really helping us.

##### Labelling

Finally, some participant reported feeling they are treated differently for having a given diagnosis, or mentioned they have seen psychiatrists talking about other patients in a negative way, which was categorised as labelling.

I met someone with schizophrenia, so I told my doctor and he said: ‘no, stay away, because he has schizophrenia, he is more severe than you, so stay away; and if he says anything, you say your doctor told you that someone with schizophrenia cannot be with someone with borderline personality disorder’, it was like, no, he has schizophrenia, with the huge label on.

#### Causes of Stigmatising Attitudes in Psychiatrists

Participants considered that the main factors related to stigmatising attitudes in psychiatrists are: desensitisation, burnout, lack of training, constant change of consultants and patients’ expectations about psychiatric treatment.

##### Desensitisation

Many Participants considered that some psychiatrists have lost their sensitivity and their ability to feel empathy towards them as they are constantly treating very unwell people. However, some perceived that this desensitisation might be intrinsic to psychiatrists’ personalities, as not every professional seems to be affected by this problem.

I guess this is normal, if you are here every day listening to people complaining about the same things all the time, then you must get used or tired of this.

##### Burnout

Work overload and burnout were considered as possible factors related to negative attitudes in psychiatrists, indeed some participants showed empathy with psychiatrists, as they considered their activities are highly demanding and potentially overwhelming. *Lack of training* was also considered an important factor, as participants mentioned that not having enough training about stigma, or even about how to treat certain disorders, along with lack of enough professional experience, could be related to negative reactions in psychiatrists.

Sometimes I think is not so much that they don’t care, I feel it might be very overwhelming to listen to what the patient is telling you…

…regarding borderline personality disorder, not everybody and not all specialists from the psychology and psychiatry area, know much about it, they just label you.

##### Constant Change of Psychiatrists

A constant change of psychiatrists was considered to cause psychiatrists to waste important time in getting to know their patients, thus creating poor therapeutic alliance, which even when this is achieved, it’s lost once doctors are relocated to new services. Participants also considered that having *unrealistic expectations about psychiatric treatment* could be considered a factor for negative or stigmatising attitudes in psychiatrists; as this can lead to patients feeling frustrated and upset if their expectations are not met, which can subsequently cause poor therapeutic alliance and unsatisfying doctor-patient relationships.

…my appointments are every three months, and it is the same, they keep changing them (psychiatrists). I get to have two or three appointments with one and then they change… they always ask the same question: ‘how do you feel? OK’; then medication and goodbye.

I had the idea that my doctor it was like a God, in the sense that she had to tell me: walk three steps here to the left or walk that way… I wanted to see her as my saviour, as my adviser, my teacher, my everything, until I realised she was only my psychiatrist.

### Interventions to Improve Psychiatrists’ Attitudes Towards People with Mental Illness

Participants recognised psychiatrists might be benefited from receiving some kind of anti-stigma intervention, and suggested two measures to improve attitudes of psychiatrists towards their own patients: raising awareness and providing training for psychiatrists.

#### Raising Awareness and Formal Training

According to participants, it is important to raise awareness about the potential consequences of psychiatrists’ attitudes, as they need to understand that patients seek help because they are ill and felt unwell not because they are seeking attention. Regarding training, participants considered this should focus on raising awareness and improving empathy, and suggested that patients should be involved in this training or should be at least more proactive when dealing with their consultants. Additionally, some participants also suggest that psychiatrists would benefit from having activities focused on distracting them from their regular psychiatric activities, for them to rest, in order to avoid desensitisation.

We don’t come here because ‘I’m so happy!’, we come here because we have a situation going on, we don’t come to receive cuddles, we understand that they are not going to hug us and so; but they need to be more conscious about one’s suffering.

Psychiatrists should keep receiving training for them to understand all the different illnesses, for them not to … stigmatise. I mean you should help patients to understand their illness, you should have some empathy.

## Discussion

The aim of this study was to explore mental health service users’ perceptions of stigma from psychiatrists and the general public in Mexico. Our results showed that, as reported in other studies, participants considered members of the general population the main source of stigma. However, one of the most important findings was that participants also considered psychiatrists to hold stigmatising attitudes towards their patients, an issue that although it had not been studied before in this population, seems to represent a barrier to optimal quality of care. Therefore, our discussion is focused on this particular issue.

### Perceptions of Stigma by Psychiatrists

Although overall participants reported satisfactory relationships with their psychiatrists, participants considered that some of psychiatrist’ attitudes could be due to stigma. The most commonly reported problems were: cold treatment and limited information about diagnoses and treatments; which concur with results from other studies (Schulze and Angermeyer 2003; Rose and Thornicroft 2010). Likewise, service users’ feel psychiatrists tend to focus on illness and medication, rather than on patients’ personal lives (Schulze and Angermeyer 2003; Buizza et al. 2007; Bonnington and Rose 2014; Hamilton et al. 2016), while psychiatrists carry out labelling has also been reported elsewhere (Angermeyer and Matchinger 2003; Lauber et al. 2006; Schulze 2007).

Participants in this study considered that stigma in psychiatrists could be a consequence of desensitisation and burnout. Although burnout has been already considered as cause of stigma in psychiatrist (Schulze 2007), many participants complaining about poor empathy from psychiatrists were part of focus groups formed by patients with borderline personality disorder. Patients who are commonly considered by psychiatrists as difficult, demanding and frustrating (Black et al. 2011; Bodner et al. 2015; Chartonas et al. 2017; Knaak et al. 2015). Therefore, is possible that this perceived desensitisation from their psychiatrists was indeed associated with psychiatrists’ negative attitudes towards patients already diagnosed with borderline personality disorder.

Lack of training about the effects of stigma and frequent changes of doctor have not previously been considered as possible causes of stigma in psychiatrists. However, some authors recommend anti-stigma interventions targeting mental health professionals that provide education about stigma (Lyons et al. 2015; Horsfall et al. 2010). Having set expectations about their treatment was considered by participants, to be in part a consequence of their own personality. Nevertheless, they considered that this could be avoided if psychiatrists explained from the beginning what their treatment involves. The media’s depiction of psychiatry may contribute to unrealistic expectations about treatment. For example, service users might expect consultations to be like a psychotherapy session.

Results from this study not only support the need for anti-stigma interventions for ‘psychiatrists, but they also highlight the need of improving psychiatrists’ knowledge about the implications of mental health stigma; and management of unconscious bias, which might be present in clinical care when a clinician classifies a patient as a member of a stereotyped group (Teal et al. 2012). The adoption of a reflective learning approach could also help improve the attitudes of psychiatrists in Mexico, as this model of learning has become popular in health care education over the last decade (Bekas 2013; Fragkos 2016); and its implementation has translated into better medical practice (Gibbs et al. 2005).

### Strengths and Limitations of the Study

A key strength of this study was the number of participants, who provided multiple points of view and accounts of their experiences. The inclusion of participants with a variety of mental illnesses provided some data that may be diagnostically specific and hence specifically relevant to the design of an intervention for psychiatrists. The inclusion of existing therapy groups of participants might have helped individuals to feel freer to speak as they were already used to sharing their experiences and opinions within the group. Having said this, including existing groups of patients can also be considered a limitation, because existing dynamic groups could have influenced individual participation or biased participants’ responses.

Although participants were assured that their participation would not have negative consequences for them, having focus groups within the hospital premises might have made participants anxious or unwilling to disclose any experience of discrimination by their psychiatrists, for fear of a negative consequence for their treatment. However, participants might have felt motivated to take part because they were not satisfied with their treatment, considering their participation as an opportunity to complain about this. The lack of inclusion of inpatients could be considered another limitation because their care differs to that received by outpatients. However, more than ten per cent of participants had been inpatients previously. Because this study was conducted only in one hospital, it is not possible to generalise our results.

## Implications of the Findings

Anti-stigma interventions are not only necessary for the general population in Mexico but also for Mexican psychiatric trainees. Interventions could be provided to medical students to reach all physicians and as negative attitudes may arise or be strengthened in this critical period of academic training. Most speciality training prioritises teaching trainees to identify symptoms and provide treatments, over addressing psychosocial problems (Bekas 2013). Therefore, specific training on the effects of mental health stigma in people’s lives; along with training focused on how to treat people with personality disorders, and how to deal with trainees’ own burnout and unconscious bias, need to be included in psychiatric curriculum to improve the quality of care and patients’ experiences.
